# A concern for ophthalmic antibiotic resistance in Bangladesh

**DOI:** 10.1002/puh2.121

**Published:** 2023-09-21

**Authors:** Md Nasir Ahmed, Mohammed Rahmatullah, Rownak Jahan, Chowdhury Alfi Afroze

**Affiliations:** ^1^ Department of Biotechnology and Genetic Engineering University of Development Alternative Dhaka Bangladesh

In 2022, Bangladesh witnessed a sudden surge in conjunctivitis infections with some hospitals reporting three times the usual patient load with conjunctivitis [1]. According to media reports, conjunctivitis, characterized by red eyes, eye pain, and irritation, may have spiked in Bangladesh since July 2022 and reached its peak by September [2]. The outbreak of conjunctivitis has been reported mainly in Chattogram, Dhaka, Sylhet, Narayanganj and Faridpur regions [3].

Conjunctivitis in most cases is caused by viral infections. In the current outbreak, nearly half of these patients are infected with human adenovirus [1]. Bangladesh has noted an outbreak of conjunctivitis during the COVID‐19 pandemic with eye‐related manifestations such as red eye, itching, watering, vision loss, acute infection and inflammation [4, 6]. Earlier outbreaks of acute viral haemorrhagic conjunctivitis had also happened in 1981 caused by enterovirus 70 (EV 70) [7].

Viral infections and viral conjunctivitis do not need antibiotics unless there is an added bacterial infection. However, in the current outbreak, topical antibiotics such as Moxifloxacin, and Chloramphenicol are freely dispensed by pharmacy retailers without the prescription of health professionals [2]. In addition, there is also rampant use of Ciprofloxacin, Dexamethasone, and Natamycin for a viral eye infection [8]. These activities are a cause of concern in terms of antimicrobial stewardship and rising antimicrobial resistance of ocular pathogens such as *Staphylococcus* spp., coagulase‐negative staphylococci, methicillin‐resistant, and *Haemophilus* spp.) in recent decades has been a concern [9]. In 2017, a study of antibiotic susceptibility on bacteria collected from infected eyes in Bangladesh, *Staphylococcus*, *Streptococcus*, and *Pseudomonas* species were resistant to Gatifloxacin, Gentamicin, Tobramycin, Cloxacillin, Ciprofloxacin, Moxifloxacin, Cefixime, and Cephalexin [10]. An increase in ophthalmic antibiotic resistance may be a major problem when indicated in cataract surgery, corneal ulcers or bacterial eye infections.

Raising public awareness regarding the judicious use of ophthalmic antibiotics is essential to avoid unprecedented ophthalmic epidemics and antimicrobial resistance burden. Regulations for self‐medication, distribution, and selling antibiotics should include ophthalmic antibiotics. Bangladesh should initiate nationwide surveillance on antibiotic resistance in ocular microorganisms.

## AUTHOR CONTRIBUTIONS

*Visualization*; *resources*; *writing – original draft*; *project administration*: Md Nasir Ahmed. *Conceptualization*; *writing – review and editing*; *supervision*: Mohammed Rahmatullah. *Validation*; *writing* – *review and editing*: Rownak Jahan. *Investigation*: Chowdhury Alfi Afroze.

## CONFLICT OF INTEREST STATEMENT

The authors have declared no conflicts of interest.

## Data Availability

Data sharing is not applicable to this article as no new data were created or analysed in this study.

## References

[1] New Age . Conjunctivitis cases on rise. New Age | The Most Popular Outspoken English Daily in Bangladesh. n.d. Accessed November 12, 2022. https://www.newagebd.net/article/182757/conjunctivitis‐cases‐on‐rise

[2] The Business Standard . As conjunctivitis surges, doctors caution against needless use of antibiotic drops. The Business Standard. 2022. Accessed November 12, 2022. https://www.tbsnews.net/bangladesh/health/conjunctivitis‐surges‐doctors‐caution‐against‐needless‐use‐antibiotic‐drops‐506030

[3] Mokbul MI , Islam AMK , Tabassum MN , Nur FB , Sharmin S . Recent incidence of infectious conjunctivitis in Bangladesh: Are we aware? Ann Med Surg. 2022:104819. 10.1016/j.amsu.2022.104819 PMC9793115 36582876

[4] Ahmed MS , Kabir MS , Bhuiyan MH . Covid‐19: Effects on eye in Bangladesh. Bangladesh Med J. 2020;49(2):14‐18. 10.3329/bmj.v49i2.55814

[5] Islam SRU , Akther T , Mahfuzullah MA , et al. Detection of SARS‐CoV‐2 RNA in conjunctival swab of a COVID‐19 patient: the first report from Bangladesh. SAGE Open Med Case Rep. 2020;8:8. 10.1177/2050313X20964103 10.1177/2050313X20964103 PMC8825635 35154766

[6] Ul Kadir SM , Moniruzzaman M , Rahman S , Nasa K , Ahmed T , Maurya RP . The impact of COVID‐19 on ophthalmic practice in Bangladesh – a cross‐sectional study. Indian J Clin Exp Ophthalmol. 2021;7(2):289‐292. 10.18231/j.ijceo.2021.059

[7] Hossain MM , Glass RI , Khan MU , Huq F , Hierholzer JC . Outbreak of enterovirus 70 conjunctivitis in Bangladesh – 1981. Trans R Soc Trop Med Hyg;77(1983):217‐218. 10.1016/0035-9203(83)90075-5 6306874

[8] Sharifa S , Anwar P , Shahida P , Abdullah MA . Prevalence of eye disease and its treatment pattern in Bangladesh: a case study of Ispahani Islamia Eye Hospital. Pharmacologyonline. 2019;3:120‐133.

[9] Asbell PA , Sanfilippo CM , Sahm DF , DeCory HH . Trends in antibiotic resistance among ocular microorganisms in the United States from 2009 to 2018. JAMA Ophthalmol;138(2020):439‐450. 10.1001/jamaophthalmol.2020.0155 PMC7146550 32271355

[10] Talukder AK , Sultana Z , Jahan I , et al. Antibiotic resistance: New challenge in the management of bacterial eye infections. Mymensingh Med J: MMJ. 2017;26(1):29‐36. 28260752

